# Supplementary material for: A unique inhibitor conformation selectively targets the DNA polymerase PolC of Gram-positive priority pathogens
Source: Nat Commun. 2025 Nov 6;16:9784. doi: 10.1038/s41467-025-65324-8 (PMC12592400; doi:10.1038/s41467-025-65324-8)
Supplement: Supplementary file 1 — Supplementary Information [file 41467_2025_65324_MOESM1_ESM.pdf]

Supplementary Information for

A unique inhibitor conformation selectively targets the DNA polymerase PolC of Gram-positive priority pathogens

Mia Urem[1,2], Annemieke H. Friggen[1], Nina Musch[1], Michael H. Silverman[3], Christopher J. Swain[3], Michael R. Barbachyn[4], Lawrence I. Mortin[3], Xiang Yu[3]; Robert J. DeLuccia[3], Meindert H. Lamers[5,6]*, Wiep Klaas Smits[1,2]*

Affiliations
[1] Leiden University Center of Infectious Diseases (LUCID), Leiden University Medical Center, Leiden, The Netherlands
[2] Centre for Microbial Cell Biology, Leiden, The Netherlands
[3] Acurx Pharmaceuticals, Inc., Staten Island, NY 10305, USA
[4] Calvin University, Grand Rapids, MI 49546, USA
[5] Department of Cell and Chemical Biology, Leiden University Medical Center, Leiden, The Netherlands
[6] NeCEN - Netherlands Centre For Electron Nanoscopy, Leiden, The Netherlands

* Corresponding authors: Meindert H. Lamers (m.h.lamers@lumc.nl) and Wiep Klaas Smits (w.k.smits@lumc.nl)

List of supplementary data:

Supplementary Figure 1. Polymerase and exonuclease activity assays.

Supplementary Figure 2. Cryo-EM processing: *E. faecium* PolC in apo and ACX-801-bound state.

Supplementary Figure 3. Cryo-EM processing: *E. faecium* PolC in IBZ- bound state.

Supplementary Figure 4. Comparison of *E. faecium* PolC with other C-family polymerases.

Supplementary Figure 5. Comparison of exonuclease domains of *E. faecium* PolC and DnaE-type polymerases.

Supplementary Figure 6. Detailed ligand interaction plot for ACX-801 and IBZ.

Supplementary Figure 7. Model of ACX-801 lowest energy conformation.

Supplementary Table 1. Cryo-EM statistics.

Supplementary Table 2. Frequency of resistance to selected ACX compounds.

Supplementary Table 3. Oligonucleotides used in this study.

Supplementary Table 4. Plasmids used in this study.

Supplementary Table 5. Strains used in this study.

Supplementary References

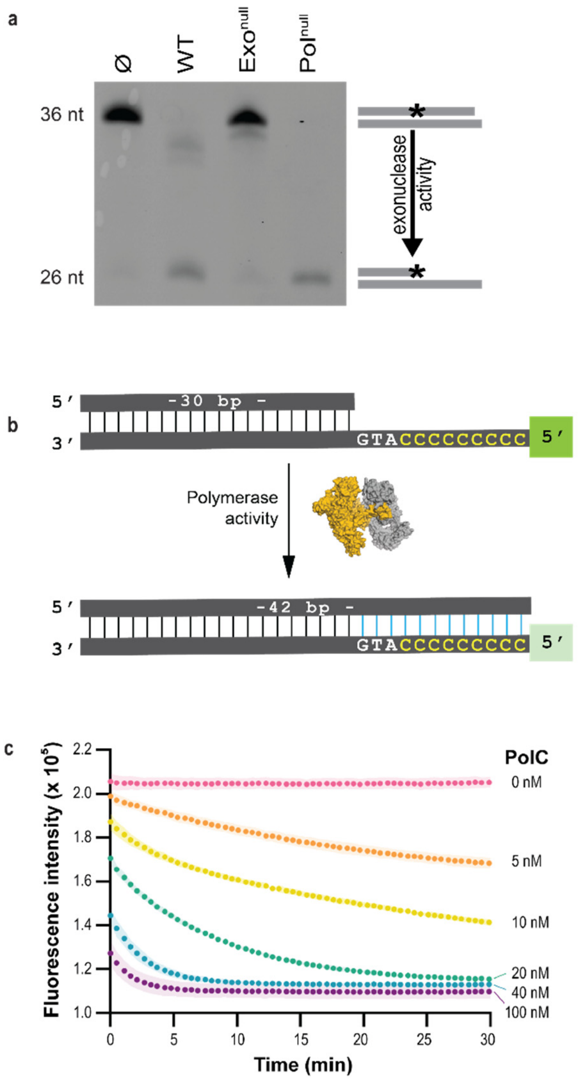

Supplementary Figure 1. Polymerase and exonuclease activity assays

a. Gel-based exonuclease assay showing exonuclease activity of wild-type *E. faecium* PolC (WT), as well as exonuclease-inactivated PolC (Exo^{null}; D431A+E433A) and polymerase-inactivated PolC (Pol^{null}; D972A+D974A). The schematic (top right) depicts the primer:template substrate with the primer one base-pair shorter than the full-length template. Below, the primer degraded up to the non-hydrolysable phosphorothioate bond (indicated by the black asterisk) is shown as the result of exonuclease activity.

b. Schematic representation of real-time DNA primer extension assay[1,2]. The 5'-end of the template contains nine cytosines followed by the fluorescein (6-FAM, bright green box). During primer extension, complementary guanines are incorporated opposite the cytosines, quenching the fluorescein signal (light green box) via photoelectron transfer. The resulting decrease in fluorescence intensity is detected in real-time as a measure of DNA synthesis as shown in Supplementary Fig. 1c.

c. Real-time primer extension curves for *E. faecium* PolC-Exo^{null} measured at varying concentrations (distinguished by colour). Without polymerase, the fluorescence signal remains constant, whereas increasing PolC concentrations accelerate primer extension. The activity is shown as an average of three replicates, with the shaded regions indicating the standard error of the mean.

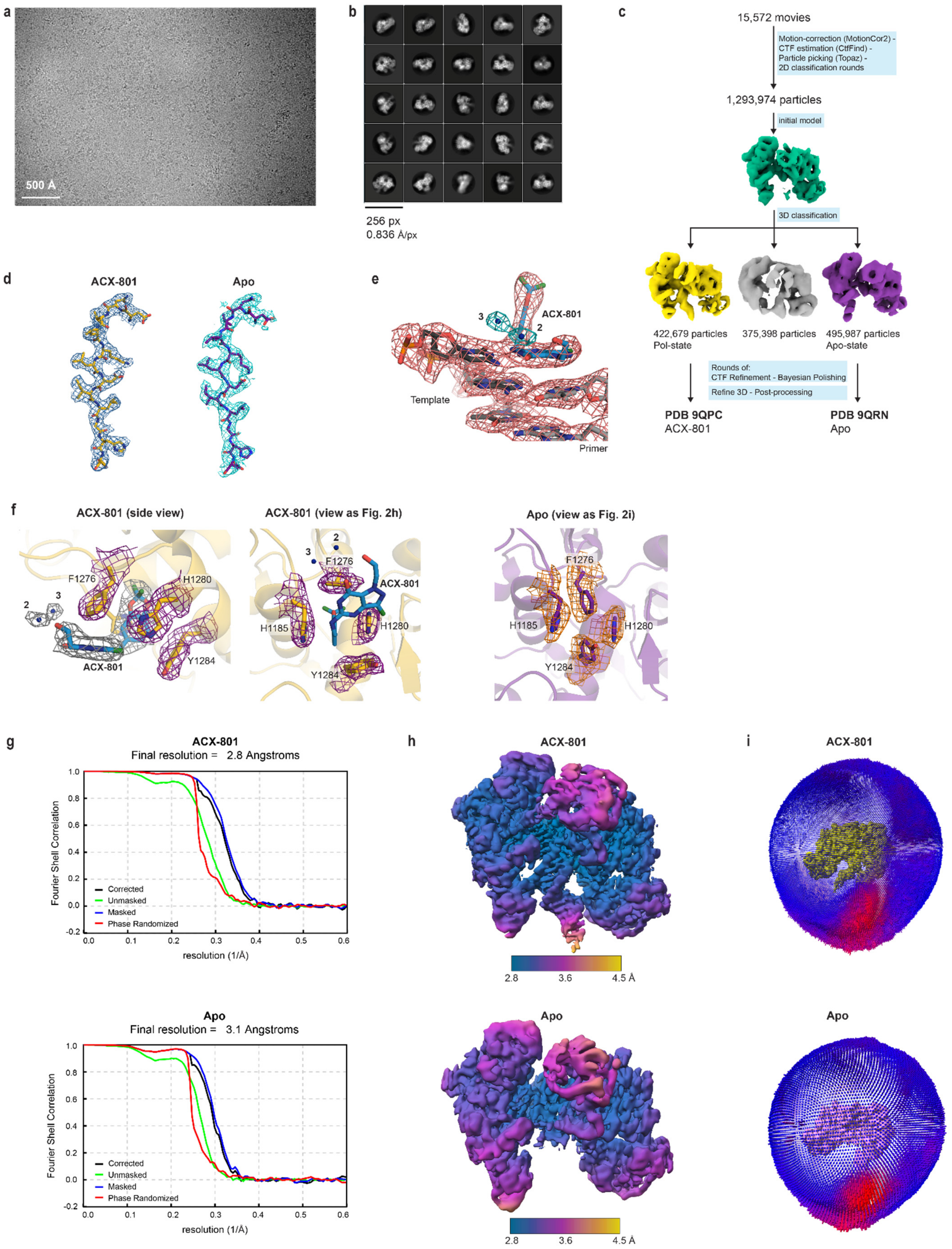
a
500 Å
b
256 px
0.836 Å/px
c
15,572 movies
Motion-correction (MotionCor2) - CTF estimation (CtfFind) - Particle picking (Topaz) - 2D classification rounds
1,293,974 particles
initial model
3D classification
422,679 particles Pol-state
375,398 particles
495,987 particles Apo-state
Rounds of: CTF Refinement - Bayesian Polishing
Refine 3D - Post-processing
PDB 9QPC ACX-801
PDB 9QRN Apo
d
ACX-801
Apo
e
ACX-801
Template
Primer
f
ACX-801 (side view)
ACX-801 (view as Fig. 2h)
Apo (view as Fig. 2i)
F1276
H1280
H1185
Y1284
g
ACX-801
Final resolution = 2.8 Angstroms
Apo
Final resolution = 3.1 Angstroms
Fourier Shell Correlation
resolution (1/Å)
Corrected
Unmasked
Masked
Phase Randomized
h
ACX-801
Apo
2.8
3.6
4.5 Å
i
ACX-801
Apo

Supplementary Figure 2. Cryo-EM data analysis of *E. faecium* PolC in the apo state (PDB 9QRN) and in complex with a DNA substrate and ACX-801 (PDB 9QPC).

a. Representative micrograph.

b. Representative 2D class averages.

c. Schematic representation of cryo-EM data processing pipeline in Relion leading to polymerase- and apo-state structures.

d. Detail of model fits to maps, showing residues D1091-D1102, is shown as a mesh contoured at 6 σ

e. Fit of ACX-801 to map is shown as a mesh contoured at 6 σ; ACX-801 stacks onto the DNA primer and base-pairs with the template strand in a position that is comparable to dGTP in the *G. kaustophilus* PolC structure (PDB 3F2B) (Supplementary Figure 4). Water molecules 2 and 3 (density map in blue for clarity), have B-factors of 25.52 $Å^2$ and 28.74 $Å^2$ (compared to an average of 49.3 $Å^2$ over the whole protein), respectively.

f. Fit of binding-pocket residues and water molecules to maps is shown as a mesh contoured at 6 σ. The side view of the pocket, relative to Figures 2g-i, highlights insertion of the R1 group into the induced pocket. Molecule colours and bottom-up viewpoint are as in Figures 2h-i. Densities for ACX-801 and water molecules 2 and 3 are shown in grey to differentiate them from residue densities (purple). For clarity, some densities are omitted in certain views.

g. Fourier Schell Correlations plots.

h. Maps coloured by local resolution.

i. Orientational distribution maps.

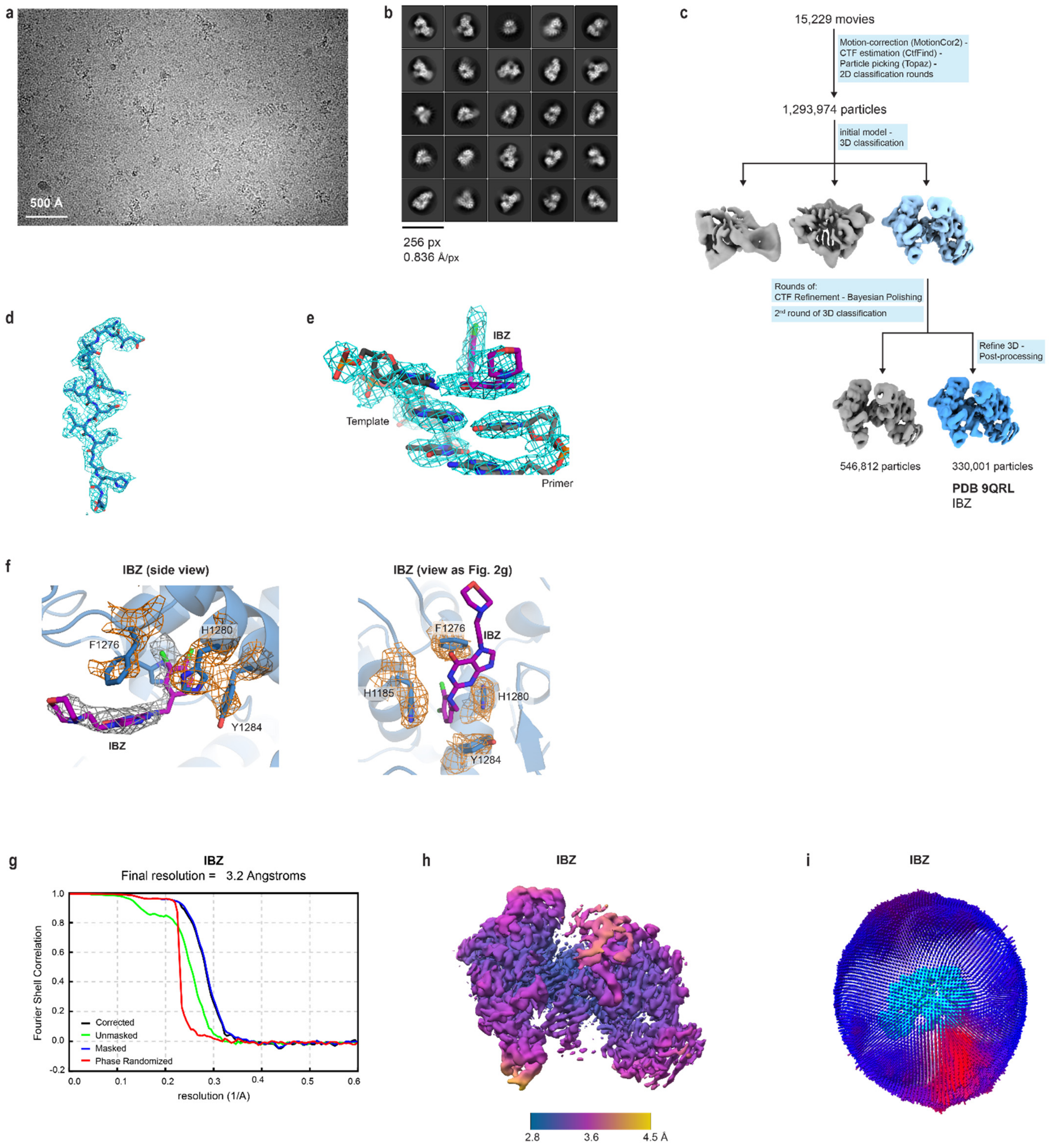

Supplementary Figure 3. Cryo-EM data analysis of *E. faecium* PolC in complex with a DNA substrate and IBZ (PDB 9QRL).

a. Representative micrograph.

b. Representative 2D class averages.

c. Schematic representation of cryo-EM data processing pipeline in Relion leading to polymerase-state structure with IBZ.

d. Detail of model fit to map, showing residues D1091-D1102, is shown as a mesh contoured at 6 σ.

e. Fit of IBZ to map, is shown as a mesh contoured at 7 σ; like ACX-801, IBZ stacks onto the DNA primer and base-pairs with the template strand in a position that is comparable to dGTP in the *G. kaustophilus* PolC structure (PDB 3F2B).
f. Fit of binding-pocket residues to map, is shown as a mesh contoured at 8 σ. The side view of the pocket, relative to Figures 2g-i and as shown in Supplementary Figure 2f, highlights insertion of the R1 group into the induced pocket. Molecule colours and bottom-up viewpoint is as in Figure 2g. The density for IBZ is shown in grey to distinguish it from the residue densities (orange). For clarity, some densities are omitted in certain views.
g. Fourier Schell Correlations plot.
h. Map coloured by local resolution.
i. Orientational distribution maps.

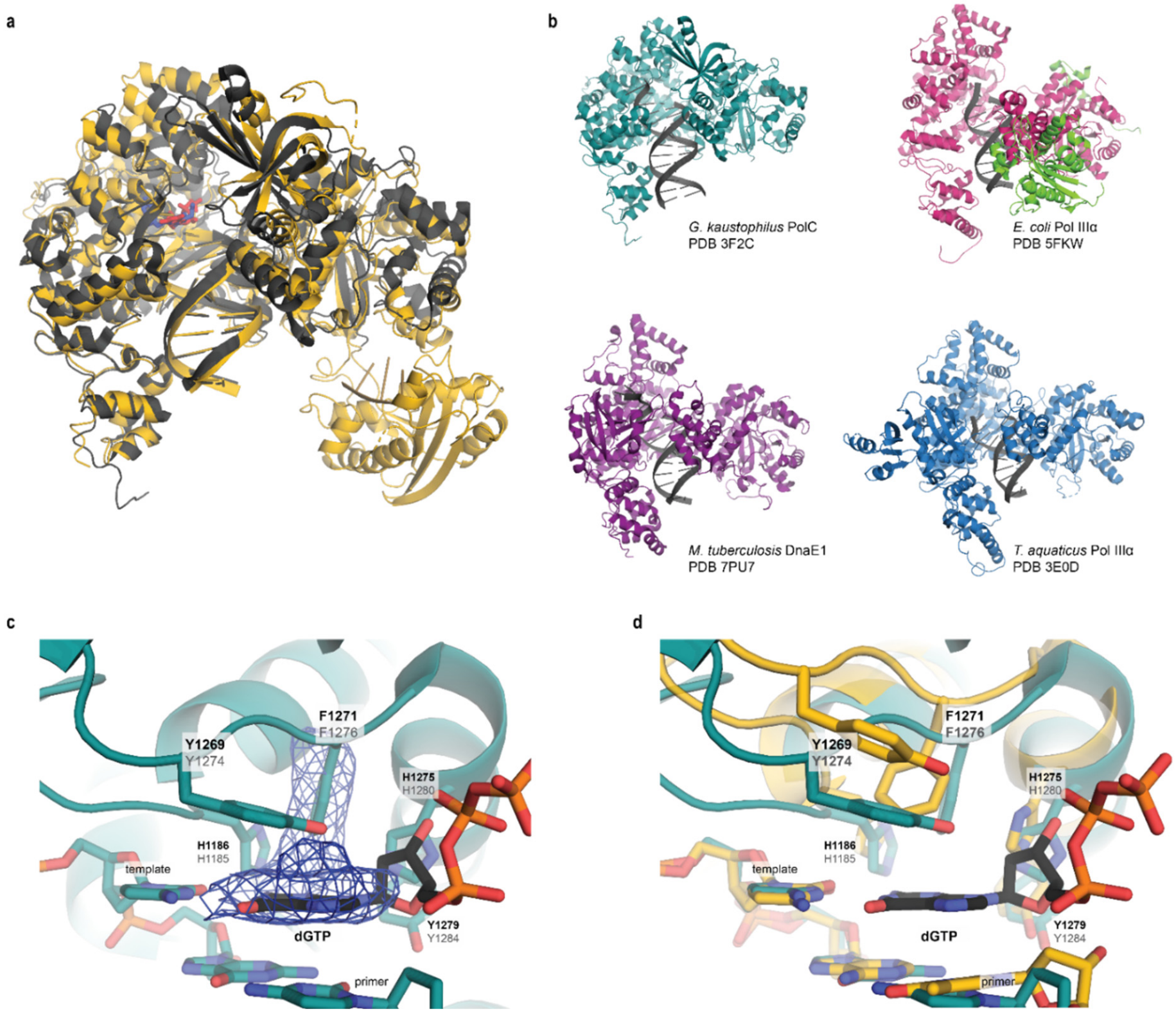

Supplementary Figure 4. Comparison of *E. faecium* PolC to other C-family polymerases.

a. Superposition of PolC structures from *E. faecium* (in yellow with blue ACX-801, PDB 9QPC) and *G. kaustophilus* (in grey with red dGTP; PDB 3F2C).

b. Comparison of C-family polymerase structures: *G. kaustophilus* PolC, *E. coli* PolIIIα (DnaE-type, PDB 5FKW), *M. tuberculosis* DnaE1 (PDB 7PU7) and *T. aquaticus* Pol IIIα (DnaE-type, PDB 3E0D). Note that the exonuclease domain is absent from *G. kaustophilus* PolC. For DnaE-type polymerases structures, additional subunits if present are not shown, with the exception of the exonuclease (subunit ε in green).

c. View of the *G. kaustophilus* PolC polymerase active site (teal) with the ACX-801 electron density (blue mesh, contoured at 6 σ; inferred from an alignment of the active sites) partially occupying the same position as the incoming dGTP (black and orange). Residues labelled in black letters are from *G. kaustophilus* and the equivalent residue in *E. faecium* is given below in grey. Note that, without the displacement of residues, ACX-801 clashes.

d. Superposed view of the DNA-bound polymerase active sites of *E. faecium* (yellow PolC and DNA, PDB 9QPC) and *G. kaustophilus* (teal PolC and DNA with incoming dGTP in black and orange). As observed for *E. faecium* apo PolC, the pocket is closed off by F1271 (F1276) and Y1269 (Y1274) contributes to tightening of the pocket toward the nucleobase of the incoming nucleotide. Note that in the *E. faecium* PolC, residue H1185 (H1275 equivalent in *G. kaustophilus* PolC) is oriented toward the induced binding pocket (for interactions with ACX-801) while in *G. kaustophilus* PolC it is interacting with the sugar of dGTP.

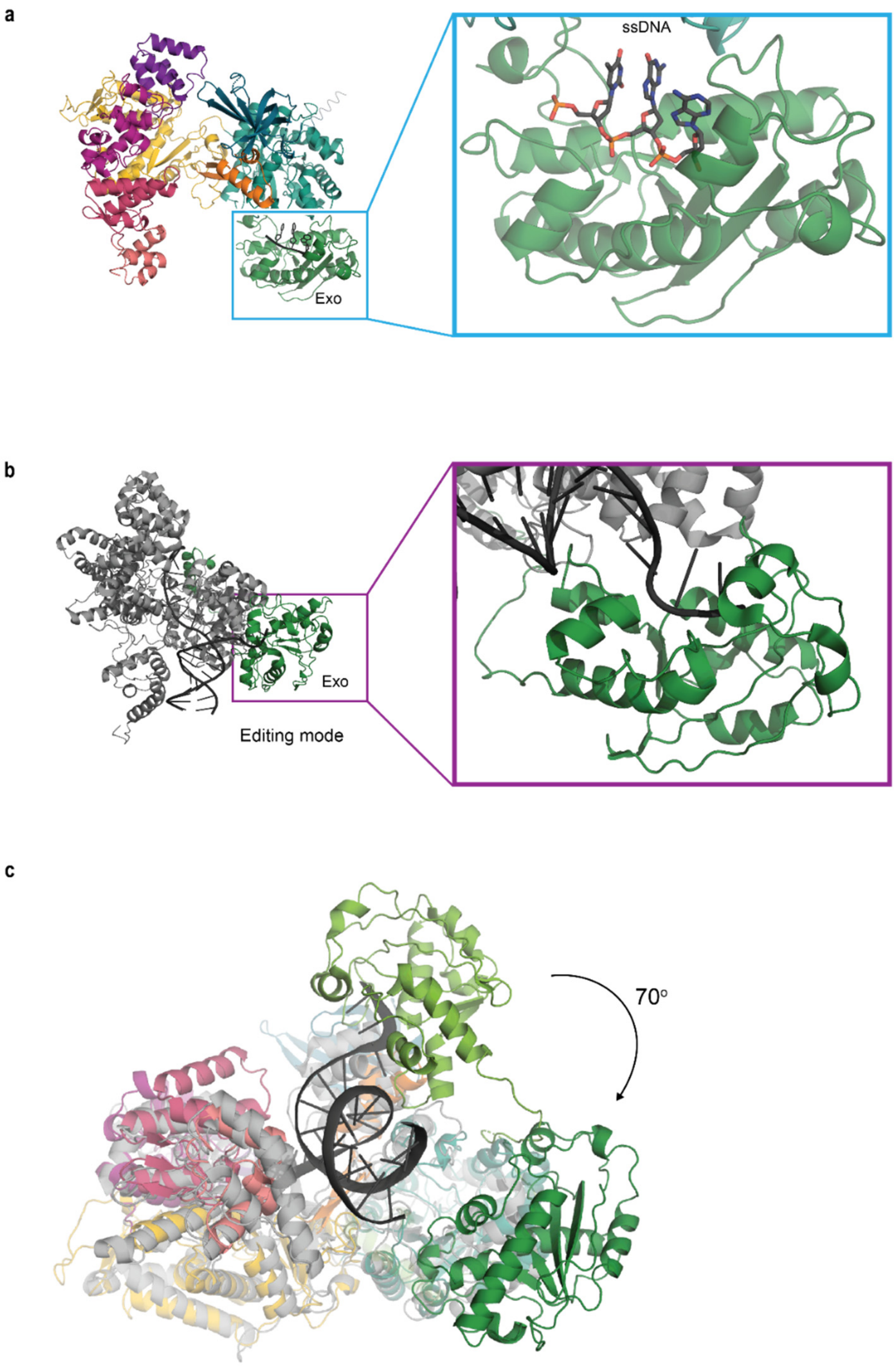

Supplementary Figure 5. Comparison of exonuclease domains of *E. faecium* PolC and DnaE-type polymerases.

a. Apo structure of exonuclease-inactivated *E. faecium* PolC (PDB 9QRN) with a close-up view of the exonuclease domain bound to a 3-nucleotide ssDNA (inset).

b. Structure of *E. coli* PolIIIα (DnaE-type, PDB 5M1S) with a close-up view of exonuclease subunit ε (green) bound to DNA (inset).

c. Bottom-view of superimposed *E. faecium* PolC (coloured as in Supplementary Fig. 5a. and Figure 1a, PDB 9QPC) and DnaE-type *E. coli* Pol IIIα polymerase (grey, PDB 5M1S). The exonuclease domain of PolC (dark green) is located below the thumb domain, whereas the exonuclease of *E. coli* Pol IIIα (separate subunit ε, in light green) is above this domain.

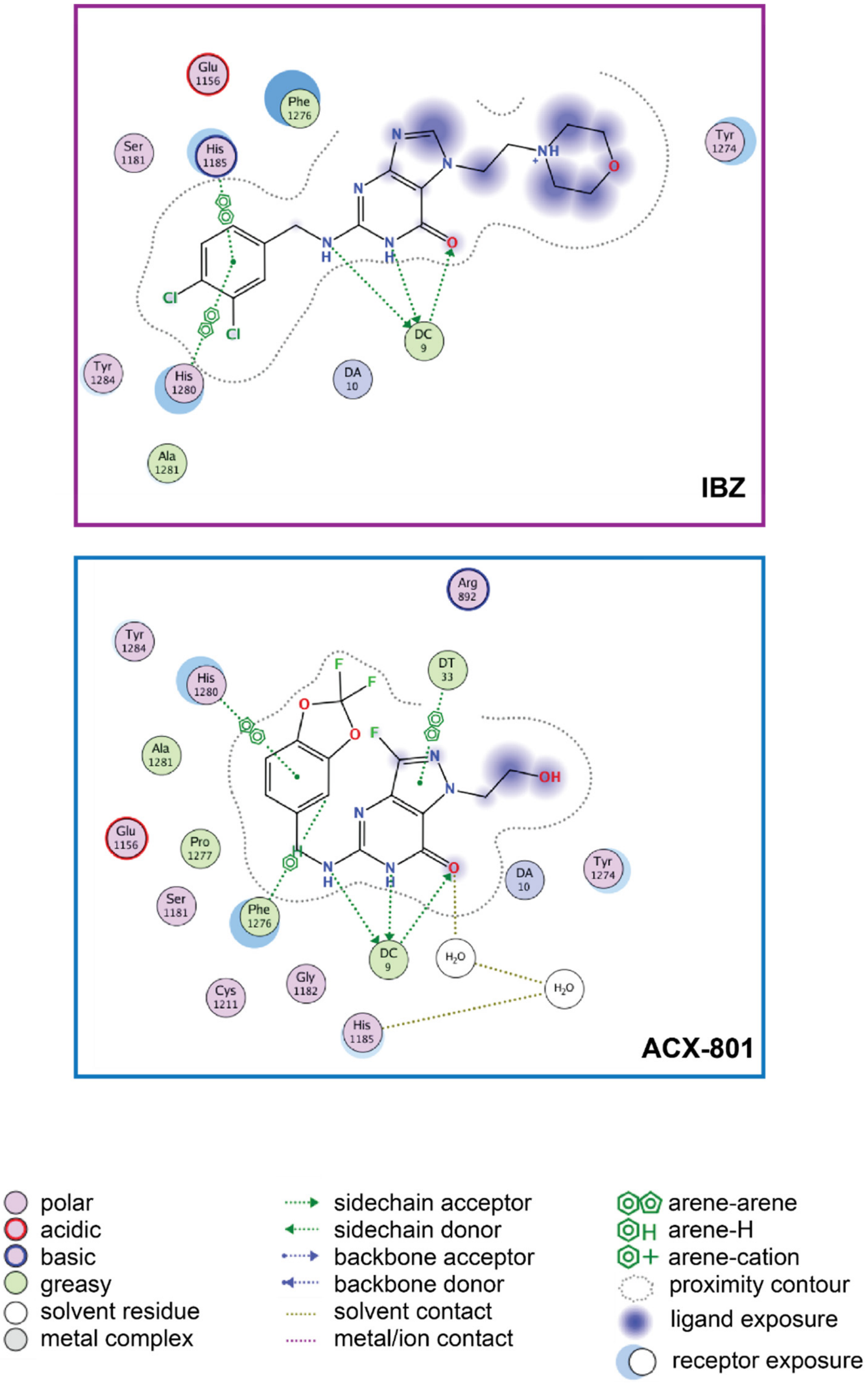

Supplementary Figure 6. Detailed ligand interaction plot for IBZ and ACX-801.

Ligand interaction plots were generated by moe2024.0601[3,4] on the basis of PDB 9QRL (IBZ) and PDB 9QRN (ACX-801).

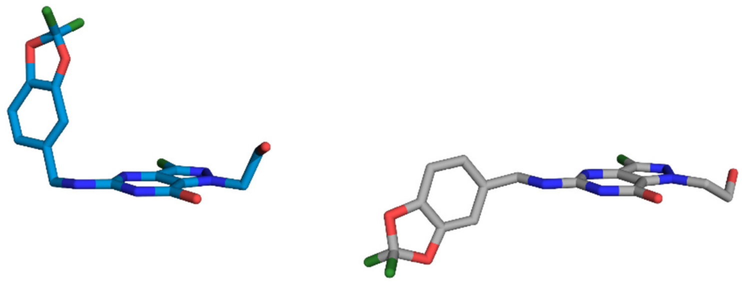

ACX-801 from PDB 9QPC

ACX-801 lowest energy conformation
from LowModeMD Search

Supplementary Figure 7. Model of ACX-801 lowest energy conformation.

The ACX-801 ligand was extracted from PDB 9QPC (blue, -88.8^{o} dihedral angle) and minimised in moe2024.0601[3,4] using MMFF94x forcefield. A conformation search was undertaken using the LowModeMD Search, a short molecular dynamics simulation using velocities with little kinetic energy on the high-frequency vibrational modes, using the default settings. The conformations were then superimposed using the pyrazolo[pyrimidine ring as the template. The lowest energy conformation (grey, 172.1^{o} dihedral angle) is shown.

Supplementary Table 1. Cryo-EM data collection, refinement and validation statistics.

	ACX- 801 (EMDB-53270) (PDB 9QPC)	Apo (EMDB-53320) (PDB 9QRN)	IBZ (EMDB-53319) (PDB 9QRL)
**Data collection and processing**			
Magnification	105k	105k	105k
Voltage (kV)	300	300	300
Electron exposure (e–/Å^2)	50	50	50
Defocus range (μm)	1.0 to 1.8	1.0 to 1.8	1.0 to 1.8
Pixel size (Å)	0.836	0.836	0.836
Symmetry imposed	C1	C1	C1
Initial particle images (no.)	1,293,974	1,293,974	2,271,326
Final particle images (no.)	422,679	495,987	330,001
Map resolution (Å)	2.8	3.1	3.2
FSC threshold	1.43	1.43	1.43
Map resolution range (Å)	2.8 to 4.5	2.9 to 4.5	3.1 to 4.9
**Refinement**			
Initial model used (PDB code)	Alphafold A0A133CXW7	9QPC	9QPC
Model resolution (Å)	2.8	3.2	3.2
FSC threshold	0.5	0.5	0.5
Map sharpening *B* factor (Å^2)	82	126	115
Model composition			
Non-hydrogen atoms	10177	8842	9160
Protein residues	1213	1113	1094
Nucleotide	27	3	23
Ligands	1	0	1
Waters	3	0	1
B factors (Å^2)			
Protein	49.3	43.9	42.9
Nucleotide	45.9	36.0	39.5
Ligand	24.3	n.a.	25.6
R.m.s. deviations			
Bond lengths (Å)	0.007	0.011	0.008
Bond angles (°)	0.900	1.381	0.967
Validation			
MolProbity score	2.89	3.08	2.82
Clashscore	25.2	23.1	21.38
Poor rotamers (%)	8.4	17.9	8.72
Ramachandran plot			
Favored (%)	95.7	96.1	95.9
Allowed (%)	4.3	3.9	4.1
Disallowed (%)	0.0	0.0	0.0

Supplementary Table 2. Frequency of resistance to selected ACX compounds.

Frequency of resistance indicates an averaged value of two replicates. Linezolid was included as a comparator in these experiments.

Concentration (mg/L)	Fold MIC	Compound	Number of reduced susceptible colonies		Frequency of resistance	
			1.3E+07 cells plated	1.3E+08 cells plated	1.3E+07 cells plated	1.3E+08 cells plated
4	2	ACX-728	TMTC	TMTC	NA	NA
8	4		6	TMTC	2.31E-07	NA
16	8		0	2	<7.69E-08	7.69E-09
32	16		0	0	<7.69E-08	<7.69E-09
1	2	ACX-641	3	98	1.15E-07	3.77E-07
2	4		0	4	<7.69E-08	1.54E-08
4	8		0	4	<7.69E-08	1.54E-08
8	16		0	0	<7.69E-08	<7.69E-09
2	2	ACX-671	4	TMTC	1.15E-07	3.77E-07
4	4		0	98	<7.69E-08	1.54E-08
8	8		0	4	<7.69E-08	1.54E-08
16	16		0	4	<7.69E-08	<7.69E-09
8	2	ACX-763	1	TMTC	7.69E-08	NA
16	4		0	0	<7.69E-08	<7.69E-09
32	8		0	0	<7.69E-08	<7.69E-09
64	16		0	0	<7.69E-08	<7.69E-09
4	2	Linezolid	TMTC	TMTC	NA	NA
8	4		TMTC	TMTC	NA	NA
16	8		TMTC	TMTC	NA	NA
32	16		TMTC	TMTC	NA	NA

* TMTC = too many to count.

Supplementary Table 3. Oligonucleotides used in this study.

Name	Sequence (5' to 3')	Description
DNASP8_42	FAM-cccccccccatgCGCACCTAAAGTTGGGAGTCCTTCGTCCTA	Real-time polymerase assay template
oAF-93	GACATAGTTTATGATACATATCAAAG	*polC* gene of *C. difficile*
oAF-94	CACAAATCATATAACCTTGATAGC	*polC* gene of *C. difficile*
oAF-95	CCAGACAAGGATTGTCCAAAG	*polC* gene of *C. difficile*
oAF-130	CAACGTACGTTGTTTTCGCTGTTGCGACCACCGGCCTTTCAG	Codon optimized *polC* gene of *E. faecium* D431A and E433A QuikChange
oAF-131	CTGAAAGGCCGGTGGTCGCAACAGCGAAAACAACGTACGTTC	Codon optimized *polC* gene of *E. faecium* D431A and E433A QuikChange
oAF-132	CACGGTGATAAGGTACCGGCCATCGCCCTGAATTTTAGTGGC	Codon optimized *polC* gene of *E. faecium* D972A and D974A QuikChange
oAF-133	GCCACTAAAATTCAGGGCGATGGCCGGTACCTTATCACCGTG	Codon optimized *polC* gene of *E. faecium* D972A and D974A QuikChange
oAF-138	CGCGTGGAACTGCACGTGC	Codon optimized *polC* gene of *E. faecium*. Sequence confirmation
oAF-139	GTAACGCGCATAACTGGTGTTC	Codon optimized *polC* gene of *E. faecium*. Sequence confirmation
oAF-140	CAACGAGGACGGTTACCTGG	Codon optimized *polC* gene of *E. faecium*. Sequence confirmation
oAF-141	CGTCCATGTAGTCAGGGATG	Codon optimized *polC* gene of *E. faecium*. Sequence confirmation
oAF-171	GTAATAATTTTATTCCTCTAGCATAC	*polC* gene of *C. difficile*
oAF-162	GTAAAAAGATTAAGTACATGTCCCCAAAAGCACATGCTGTTGC	*polC* gene of *C. difficile* F1258S QuikChange
oAF-163	GCAACAGCATGTGCTTTTGGGGACATGTACTTAATCTTTTTAC	*polC* gene of *C. difficile* F1258S QuikChange
oAF-164	GTAAAAAGATTAAGTACATGATCCCAAAAGCACATGCTGTTGC	*polC* gene of *C. difficile* F1258I QuikChange
oAF-165	GCAACAGCATGTGCTTTTGGGATCATGTACTTAATCTTTTTAC	*polC* gene of *C. difficile* F1258I QuikChange
oAF-174	GTACATGTTCCCAAAAGCACATACTGTTGCTTATGTAATGACATC	*polC* gene of *C. difficile* A1263T QuikChange
oAF-175	GATGTCATTACATAAGCAACAGTATGTGCTTTTGGGAACATGTAC	*polC* gene of *C. difficile* A1263T QuikChange
oAF-176	CATGTTCCCAAAGGCTCACACCGCCGCCTATGTTCTG	Codon optimized *polC* gene of *E. faecium* A1281T QuikChange
oAF-177	CAGAACATAGGCGGCGGTGTGAGCCTTTGGGAACATG	Codon optimized *polC* gene of *E. faecium* A1281T QuikChange
oAF-182	AGGCGTGTCTCAAGAGCAG	Codon optimized *polC* gene of *E. faecium*. Sequence confirmation
oAF-196	CTAAGATTAAGTACATGTTACCAAAGGCTCACGCCG	Codon optimized *polC* gene of *E. faecium* F1276L QuikChange
oAF-197	CGGCGTGAGCCTTTGGTAACATGTACTTAATCTTAG	Codon optimized *polC* gene of *E. faecium* F1276L QuikChange
oJdE015	GCTCTAAGATTAAGTACATGTCCCCAAAGGCTCACGCCGCCGC	Codon optimized *polC* gene of *E. faecium* F1276S QuikChange
oJdE016	GCGGCGGCGTGAGCCTTTGGGGACATGTACTTAATCTTAGAGC	Codon optimized *polC* gene of *E. faecium* F1276S QuikChange
oMU014	TAGGACGAAGGACTCCCAACTTTAGGTGCG	Real-time polymerase assay primer
oMU017	TAGGACGAAGGACTCCCAACTTTAGGTGCGCAT	CryoEM DNA substrate primer
oMU029	FAM-GGAGTAGTACTAGGACGAAGGACTC*Ttgtaagctag	Gel-based exonuclease assay primer with phosphorothioate (*) to block exonuclease activity; 1 shorter than full-length template
oMU039	cccccccccATGCGCACCTAAAGTTGGGAGTCCTTCGTCCTA	CryoEM DNA substrate template
oNM-001	GATCTGGTACCGCAGCGTATTCCAATGGCTGG	*polC* promoter of *C. difficile*; *Kpn*I restriction site
oNM-003	GATCTGGATCCGCTGTCTCACTCTTAGTTTCTGC	*polC* gene of *C. difficile*; *BamH*I restriction site
oWKS-135	GCGAAATTAATACGACTCACTATAGG	pET28b backbone. Sequence confirmation
oWKS-136	CAGCCAACTCAGCTTCCTTTC	pET28b backbone. Sequence confirmation
oWKS-1070	GTCTTGGATGGTTGATGAGTAC	Chromosomal *gluD*
oWKS-1071	TTCCTAATTTAGCAGCAGCTTC	Chromosomal *gluD*
oWKS-1240	CACCTCCTTTTTGACTTTAAGCCTACGAATACC	pAP24 backbone
oWKS-1241	CACCGACGAGCAAGGCAAGACCG	pAP24 backbone
oWKS-1242	CTGGACTTCATGAAAAACTAAAAAAAATATTG	pAP24 backbone
oWKS-1387	CAGATGAGGGCAAGCGGATG	*traJ* in pAP24
oWKS-1388	CGTCGGTGAGCCAGAGTTTC	*traJ* in pAP24
oWKS-1389	GCCACATAAGCACTCAAAGG	*repA* in pAP24
oWKS-1390	CCCCAAATTACTGCCATGGT	*repA* in pAP24
Temp-Phospho-NoMis	GCTAGCTTACAagagtccttcgtcctagtactactcc	Gel-based polymerase and exonuclease assay template
61: 37/26 C T Phospho	FAM-GGAGTAGTACTAGGACGAAGGACTC*T	Gel-based polymerase assay primer with phosphorothioate (*) to block exonuclease activity

Supplementary Table 4. Plasmids used in this study.

Plasmid	Relevant features
pAF365	Expression plasmid, codon optimized *E. faecium polC*; derived from pET28b
pAF386	Expression plasmid, codon optimized *E. faecium polC*$^{p.D431A/E433A}$; derived from pAF365
pAF387	Expression plasmid, codon optimized *E. faecium polC*$^{p.D972A/D974A}$; derived from pAF365
pAF477	Expression plasmid, codon optimized *E. faecium polC*$^{p.D431A/E433A/F1276L}$; derived from pAF386
pAF478	Expression plasmid, codon optimized *E. faecium polC*$^{p.D431A/E433A/F1276S}$; derived from pAF386
pAF430	Expression plasmid, codon optimized *E. faecium polC* $^{p.A1281T}$; derived from pAF365
pAF433	Expression plasmid, codon optimized *E. faecium polC* $^{p.D431A/E433A/A1281T}$; derived from pAF430
pAF479	Expression plasmid, codon optimized *E. faecium polC* $^{p.D431A/E433A/D1103Y}$; derived from pAF386
pAF480	Expression plasmid, codon optimized *E. faecium polC* $^{p.D431A/E433A/D972A/D974A}$; derived from pAF386
pAP24[5]	Cloning vector; *tetR*; P*tet-sluc*^{opt}; *catP*; *traJ*; *repA*
pNM2001	P*polC-polC*; derived from pAP24
pNM2006	P*polC-polC*$^{p.F1258L}$; derived from pAP24
pNM2023	P*polC-polC*$^{p.F1258I}$; derived from pAP24
pNM2030	P*polC-polC*$^{p.A1263T}$; derived from pAP24
pNM2044	P*polC-polC*$^{p.F1258S}$; derived from pAP24

Supplementary Table 5. Bacterial strains used in this study.

Strain	Relevant features
E. coli	
AF365	DH5α/pET28-VRE-PolC (Twist); kan^R
AF386	DH5α/pAF386; kan^R
AF387	DH5α/pAF387; kan^R
AF477	DH5α/pAF477; kan^R
AF478	DH5α/pAF478; kan^R
AF430	DH5α/pAF430; kan^R
AF433	DH5α/pAF433; kan^R
AF479	DH5α/pAF479; kan^R
AF480	DH5α/pAF480; kan^R
Rosetta(DE3) pLysS	Protein expression strain
CA434	Conjugation donor strain; HB101 carrying R702 plasmid; *kan*
DH5α	Cloning strain
NM2001	DH5α/pNM2001; cam^R
NM2006	DH5α/pNM2006; cam^R
NM2023	DH5α/pNM2023; cam^R
NM2030	DH5α/pNM2030; cam^R
NM2044	DH5α/pNM2044; cam^R
C. difficile	
NM1000/ WKS1833	Laboratory stocks of strain 630Δ*erm*[6]
NM1001/WKS2174	Obtained by growth of WKS1833 on 8 µg/mL of IBZ; contains *polC*^{pF1276L} mutation
NM1048	630Δ*erm*/pNM2001; $thiam^R$
NM1053	630Δ*erm*/pNM2006; $thiam^R$
NM1068	630Δ*erm*/pNM2023; $thiam^R$
NM1074	630Δ*erm*/pNM2030; $thiam^R$
NM1077	630Δ*erm*/pNM2044; $thiam^R$
NM1080	630Δ*erm*/pAP24; $thiam^R$

Supplementary References

1 Toste Rego, A., Holding, A. N., Kent, H. & Lamers, M. H. Architecture of the Pol III-clamp-exonuclease complex reveals key roles of the exonuclease subunit in processive DNA synthesis and repair. *EMBO J* **32**, 1334-1343 (2013). https://doi.org:10.1038/emboj.2013.68

2 Song, C., Zhang, C. & Zhao, M. Singly labeled smart probes for real-time monitoring of the kinetics of dNTP misincorporation and single nucleotide extension in DNA intra-molecular polymerization. *Biosensors and Bioelectronics* **25**, 301-305 (2009). https://doi.org:10.1016/j.bios.2009.07.002

3 Clark, A. M. & Labute, P. 2D depiction of protein-ligand complexes. *J Chem Inf Model* **47**, 1933-1944 (2007). https://doi.org:10.1021/ci7001473

4 Clark, A. M., Labute, P. & Santavy, M. 2D structure depiction. *J Chem Inf Model* **46**, 1107-1123 (2006). https://doi.org:10.1021/ci050550m

5 Oliveira Paiva, A. M., Friggen, A. H., Hossein-Javaheri, S. & Smits, W. K. The Signal Sequence of the Abundant Extracellular Metalloprotease PPEP-1 Can Be Used to Secrete Synthetic Reporter Proteins in *Clostridium difficile*. *ACS Synth Biol* **5**, 1376-1382 (2016). https://doi.org:10.1021/acssynbio.6b00104

6 van Eijk, E. *et al.* Complete genome sequence of the *Clostridium difficile* laboratory strain 630Deltaerm reveals differences from strain 630, including translocation of the mobile element CTn*5*. *BMC Genomics* **16**, 31 (2015). https://doi.org:10.1186/s12864-015-1252-7
