# Supplementary material for: A unique inhibitor conformation selectively targets the DNA polymerase PolC of Gram-positive priority pathogens
Source: Nat Commun. 2025 Nov 6;16:9784. doi: 10.1038/s41467-025-65324-8 (PMC12592400; doi:10.1038/s41467-025-65324-8)
Supplement: Supplementary file 2 — Description of Additional Supplementary Files [file 41467_2025_65324_MOESM2_ESM.docx]

**Description of Additional Supplementary Files**

**Supplementary Movie:** Movement of F1276 of *E. faecium* PolC. The movie demonstrates the residue movements required to form the induced pocket that accommodates inhibitor binding, here shown for ACX-801. The inhibitor binding pocket is shown from the same viewpoint as in Figures 2g-i.
